# Supplementary figures and images for: Tel1 is recruited at chromosomal loop/axis contact sites to modulate meiotic DNA double-strand breaks interference
Source: PLoS Genet. 2025 Nov 17;21(11):e1011904. doi: 10.1371/journal.pgen.1011904 (PMC12622785; doi:10.1371/journal.pgen.1011904)

Figure S1

a

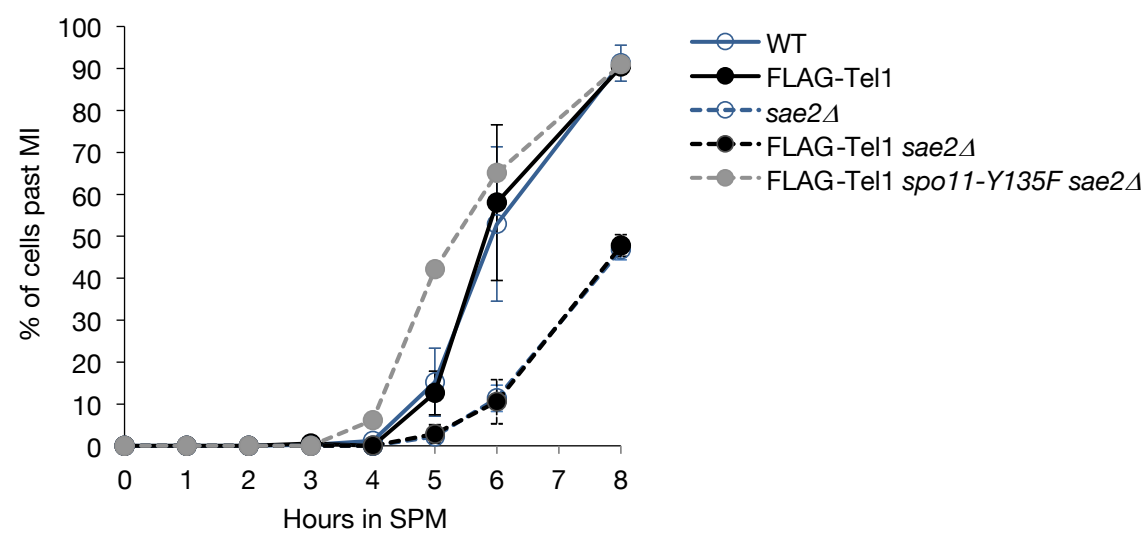

b

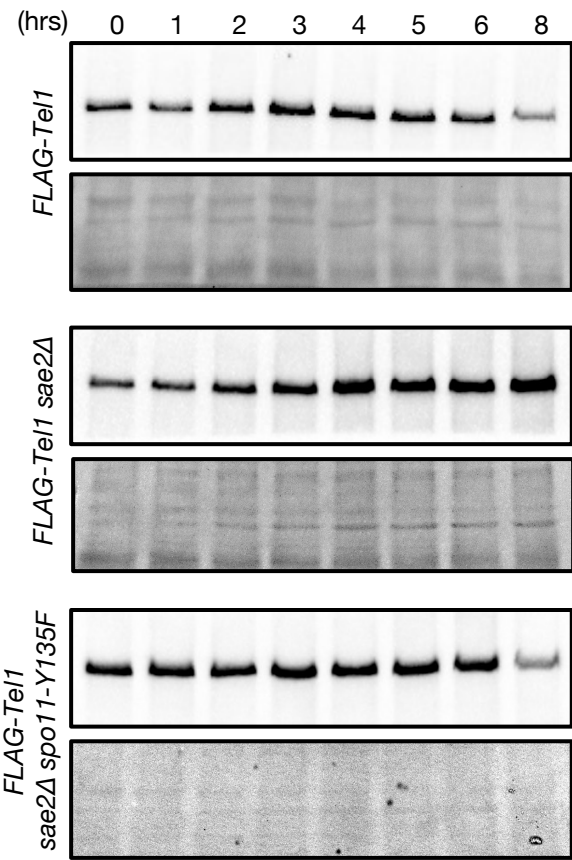

c

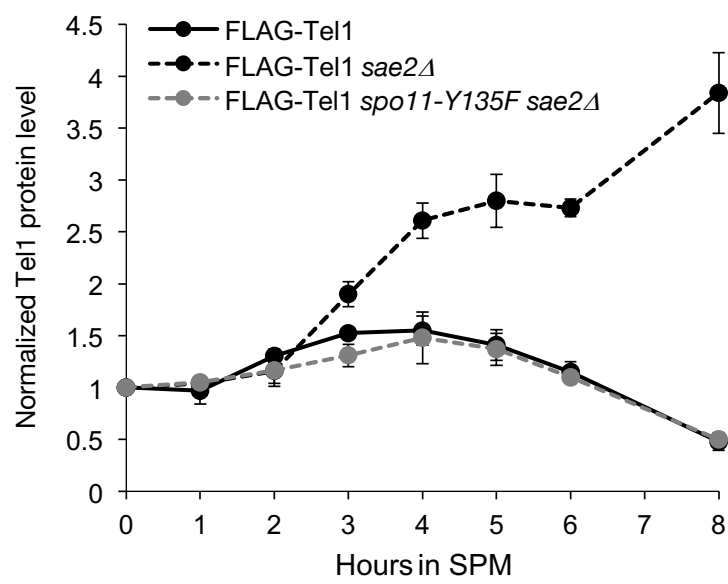

d

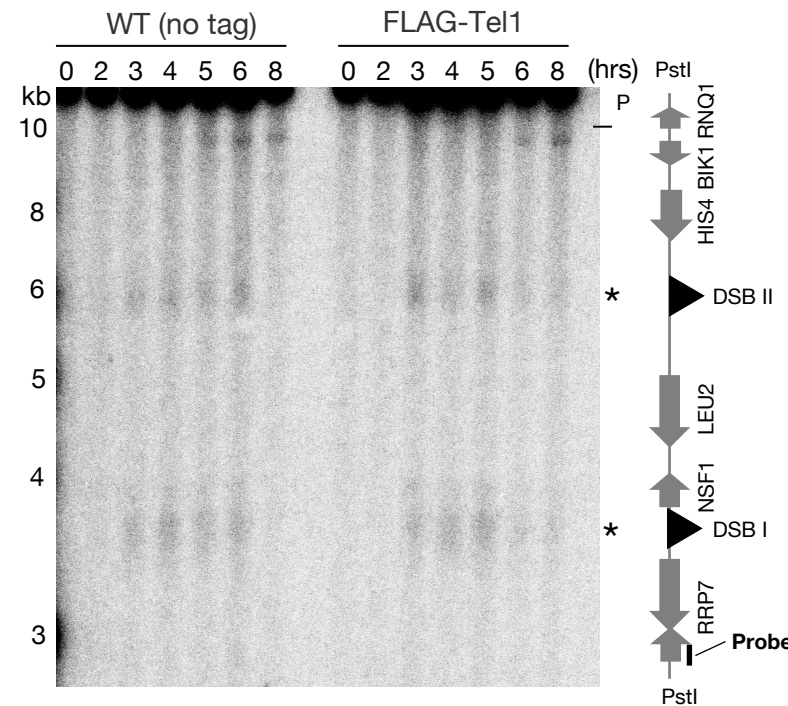

e

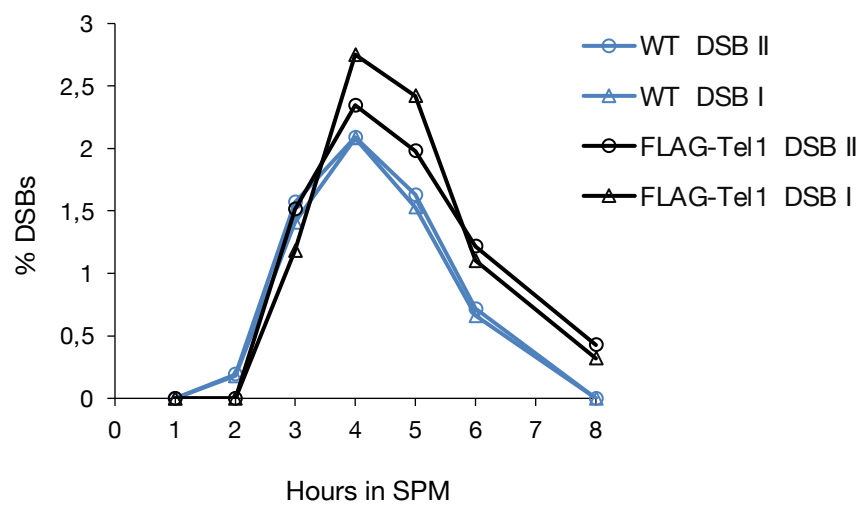

Supplement: S1 Fig — a, Meiotic progression as the percentage of cells with 2 (MI) or 4(MII) nuclei at indicated times after entering meiosis. About 200 cells were scored by DAPI staining for each time point. b, Immunoblot of meiotic TCA extracts at indicated time-points probed with anti-FLAG antibody. Bottom panels: ponceau staining. c, Quantification of immunoblots (n = 3) as per (b). Signal normalization was performed against ponceau staining before standardisation to time 0 h. d, Southern blot of PstI digested gDNA hybridized with a probe located on the MRX2 open-reading frame as indicated. P, parental DNA. DSB signals are marked with an asterisk. e, Quantification of DSBs in d (n = 1). (PDF) [file pgen.1011904.s001.pdf]

Figure S2

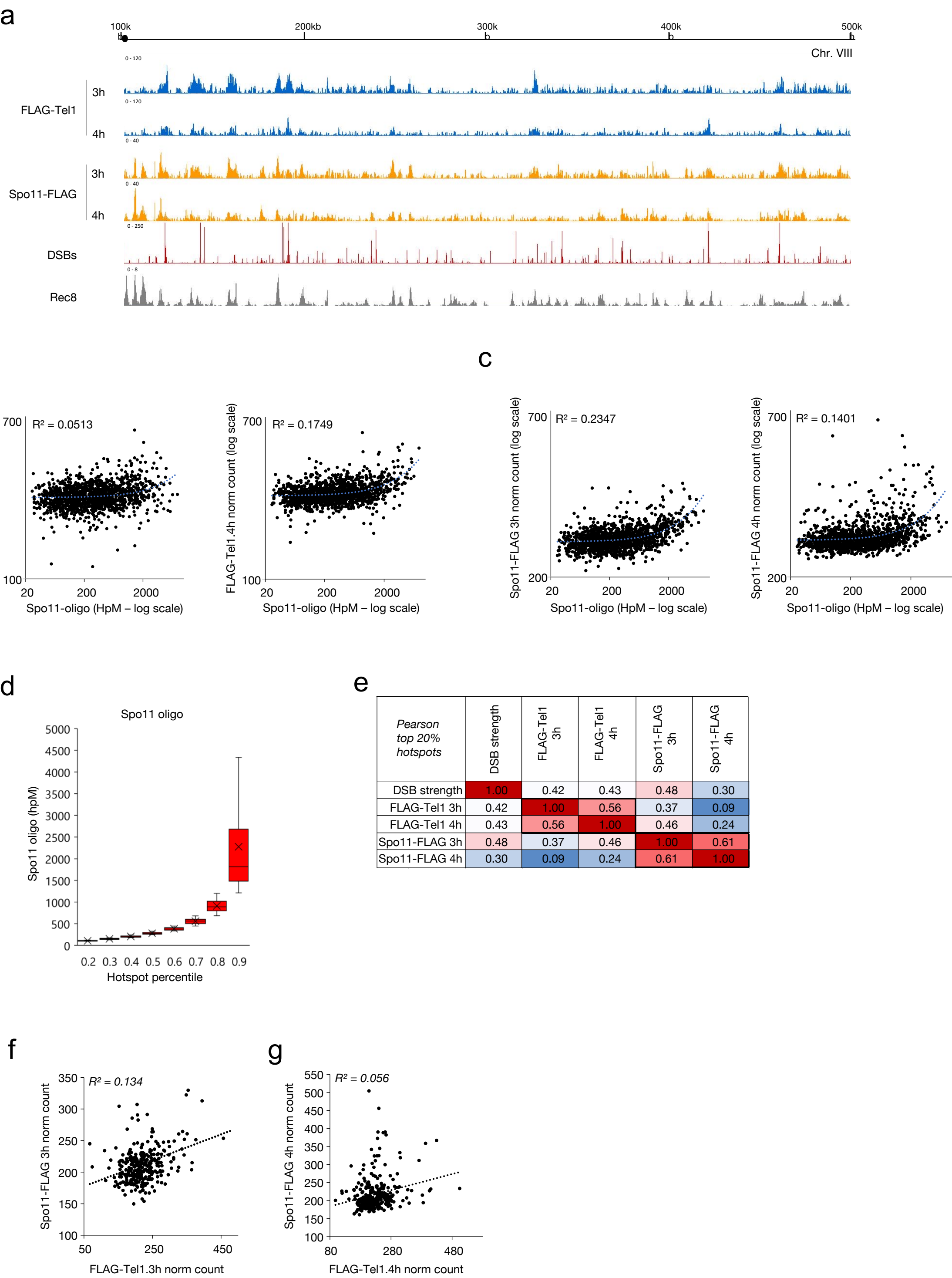

Supplement: S2 Fig — a, Read coverage of FLAG-Tel1 (blue), Spo11-FLAG (orange) at 3 and 4 h in a large region of ChrVIII. DSBs are represented by Spo11-oligos (red) and axis by Rec8-HA ChIP-seq (grey). Horizontal axis: position on chromosome VIII. Vertical axis: Read coverage for each ChIP-seq subtracted from the input signal for Tel1 and Spo11. ChIP-seq for Tel1 was performed in a wild-type background. ChIP-seq for Rec8 (GSE69231) and Spo11 (GSE52863) as well as Spo11-oligos (GSE26449) were previously performed by Sun et al. and Pan J, et al., respectively, also in a wild-type background [8,53]. b, Scatter plot between Tel1 signals at 3 and 4 h in SPM (log of normalised count over 1500pb regions centered on hotspots), and DSB signals (Spo11-oligos – log of HpM: hits per million mapped reads). c, Scatter plot between Spo11 signals at 3 and 4 h in SPM (log of normalised count over 1500pb regions centered on hotspots), and DSB signals (Spo11-oligos – log of HpM: hits per million mapped reads). d, Boxplots of Spo11-oligos (HpM) partitioned by DSB strength percentile (Spo11-oligos HpM). e, Table of Pearson correlation on the top 20% hotspots (n = 296) between DSBs (Spo11-oligos HpM), Tel1 (normalised count over 1500pb regions centered on hotspots) and Spo11 (normalised count over 1500pb regions centered on hotspots) at 3 and 4 h in SPM, relative to each other’s. f,g, Scatter plot between Spo11 and Tel1 signals (log of normalised count over 1500pb regions centered on top 20% hotspots) at 3 h (f) and 4 h (g) in SPM. R² Indicate the coefficient of determination. (PDF) [file pgen.1011904.s002.pdf]

Figure S3

a

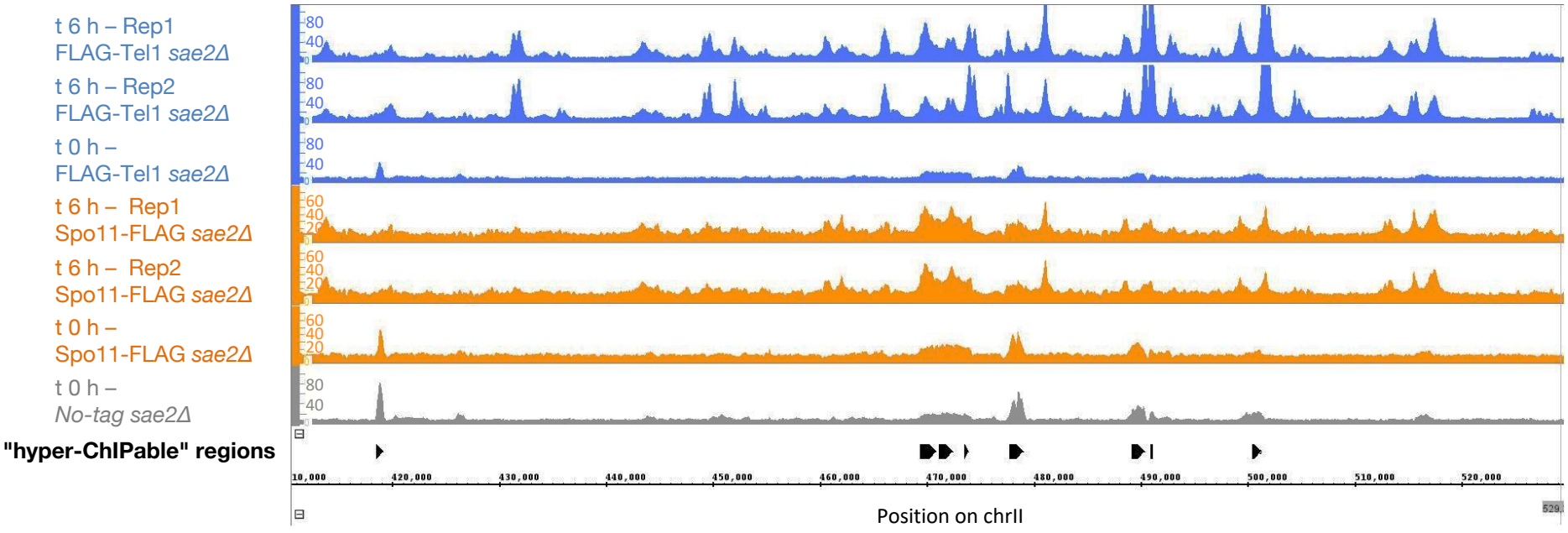

b

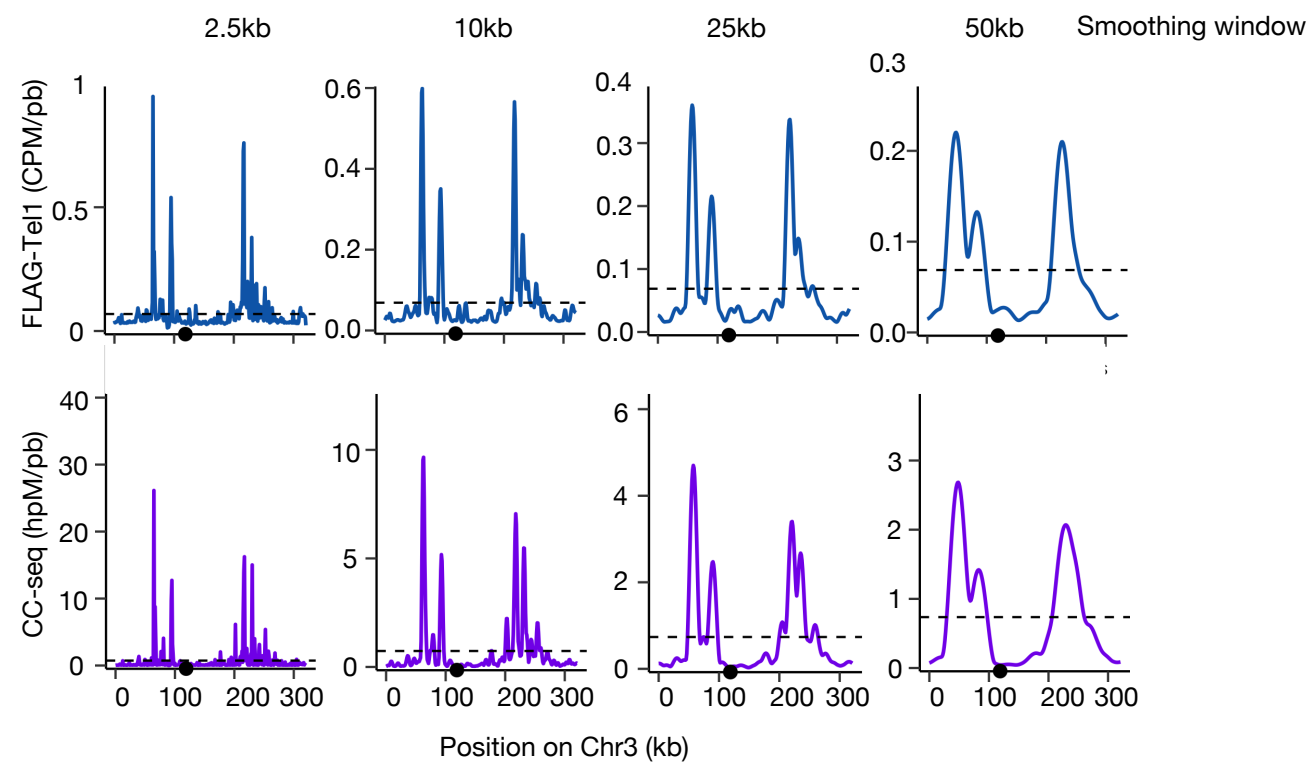

c

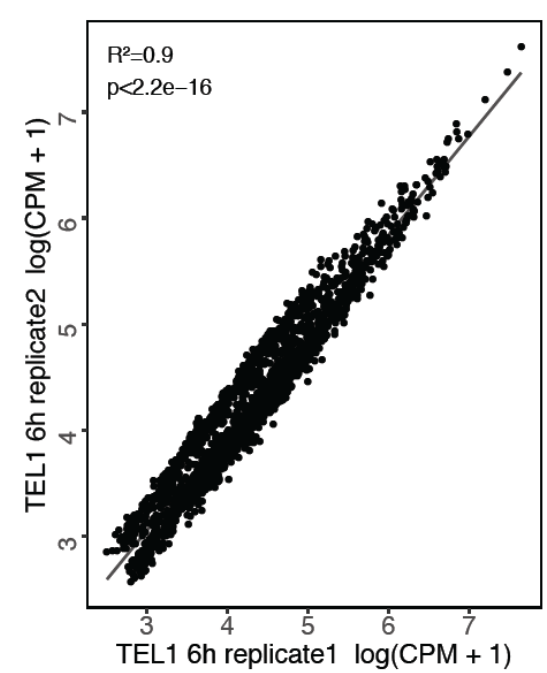

d

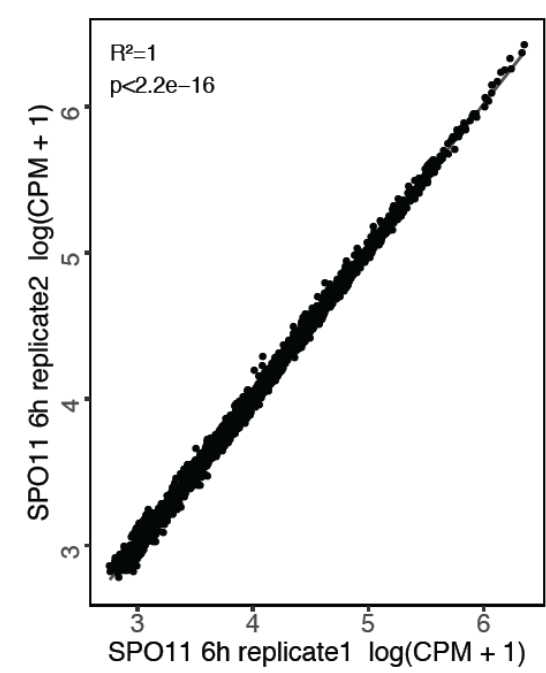

e

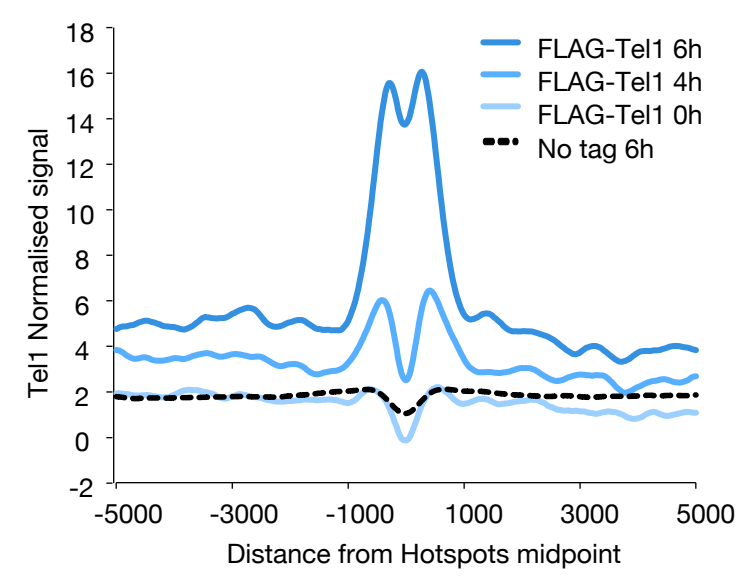

f

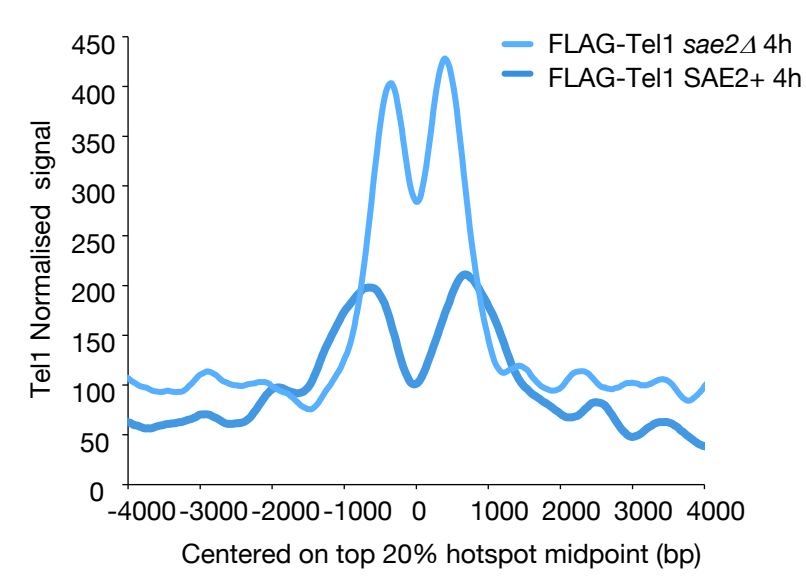

Supplement: S3 Fig — a, FLAG-Tel1 (blue) and Spo11-FLAG (orange) raw coverage signal in a sae2Δ background. Note that a similar signal is observed in the two pre-meiotic (0h) samples and the untagged sae2Δ control strain. “High-chippable” regions are peaks found in pre-meiotic and untagged samples (see Methods - Peak calling/High-ChIPable regions). b, Spatial and quantitative agreement of Tel1 signal (upper panels) with DSB signal determined by CC-seq (lower panels). The ChIP-seq profile is shown as an example on Chr3, but Tel1 and CC-seq signals agree well on all chromosomes. The position of the centromere is indicated with the black circle on the horizontal axis. Signal values are plotted after smoothing with an increasing sliding Hann window size as indicated. c, d, Correlation between biological replicates (exp2, 6 h). Tel1 (c) and Spo11 (d) using the average signals in individual 100-bp non-overlapping genomic windows. Coefficient of determination (R²) and associated p-value were determined using linear model fitting function (lm). e, FLAG-Tel1 read densities (RPKM per 20-bp bins) ±5 kb from DSBs midpoint in a sae2Δ background, from exp1 at 0h (blue light line), 4 h (blue medium line) and 6 h (blue dark line) in SPM. Dashed black line represent the profile of non-specific signal (no-tag). f, FLAG-Tel1 read densities (RPKM per 20-bp bins) ±4 kb from the top 20% hotspots midpoint, in a sae2Δ background at 4h (blue light line) versus WT background at 4h (blue dark line). (PDF) [file pgen.1011904.s003.pdf]

Figure S4

a

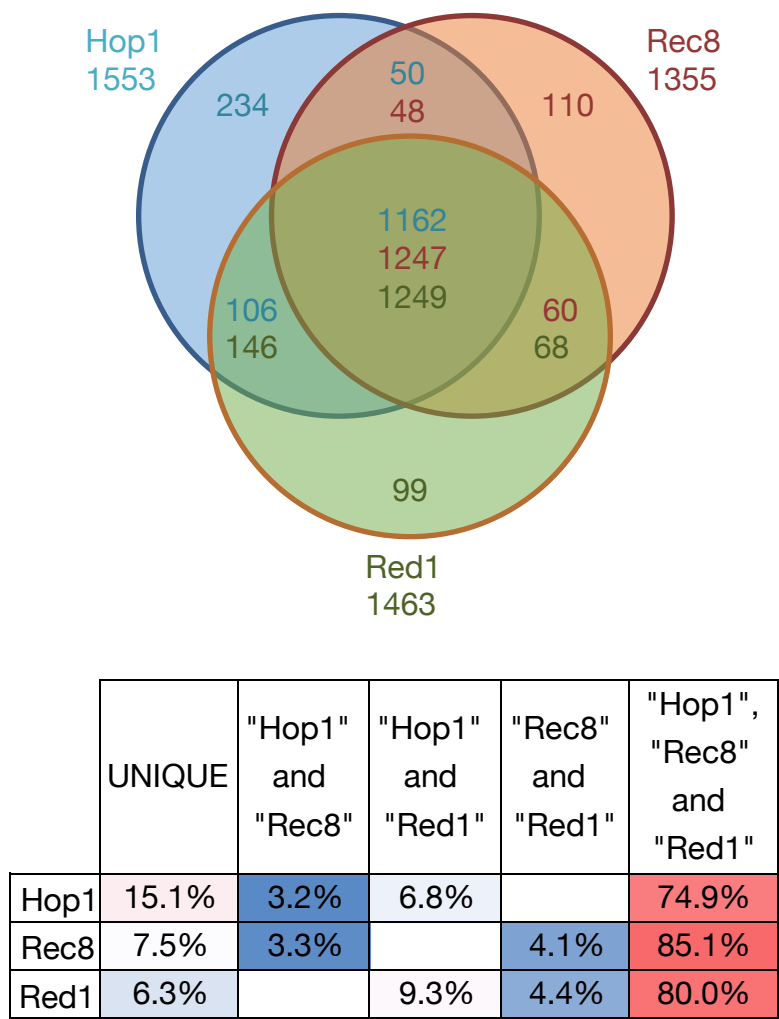

b

| Sites | Axis | DSB  | Tel1 | Spo11 |
|-------|------|------|------|-------|
| Axis  | 1357 | 789  | 1468 | 1427  |
| DSB   | 535  | 3601 | 1818 | 1681  |
| Tel1  | 1032 | 2143 | 3103 | 2126  |
| Spo11 | 1177 | 2162 | 2504 | 3042  |
| alone | 114  | 910  | 230  | 489   |

d

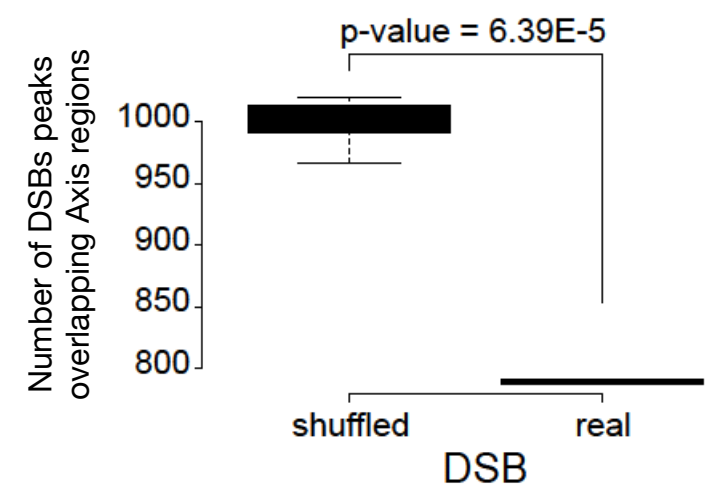

c

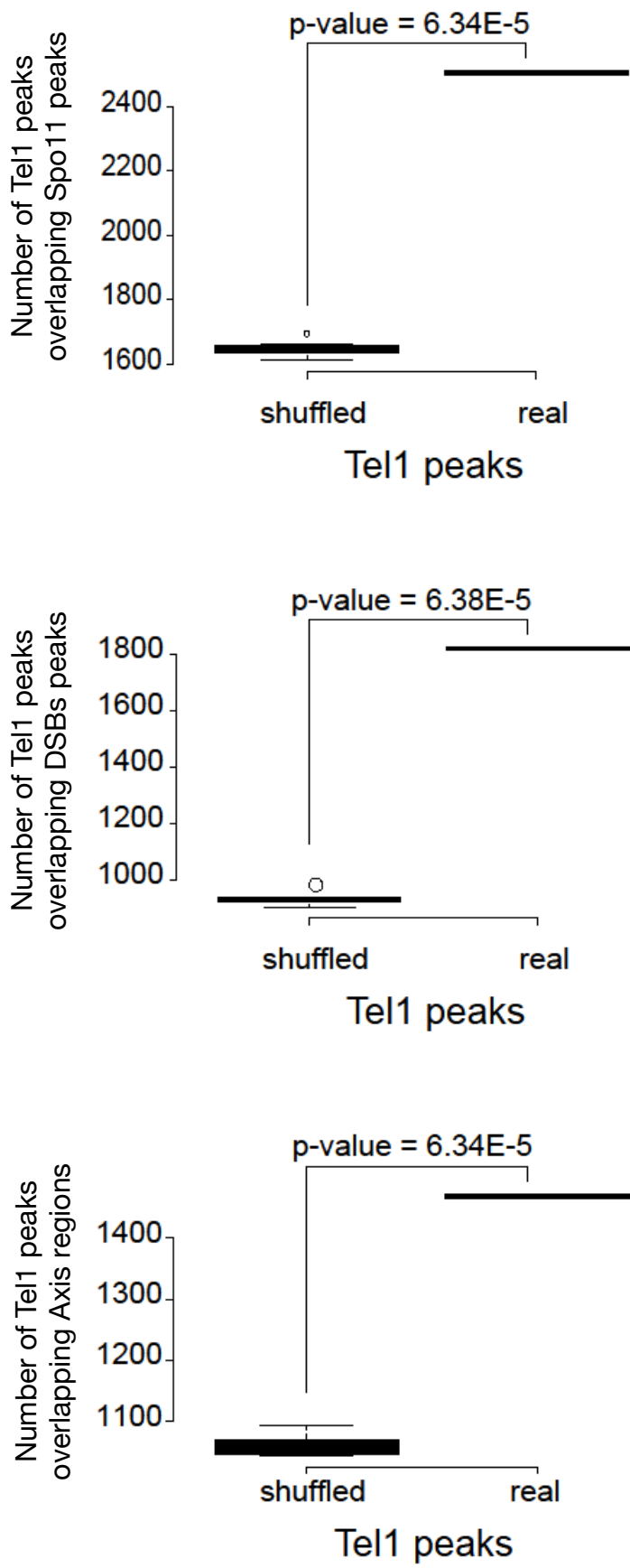

Supplement: S4 Fig — a, Venn diagrams showing the overlap between Hop1, Rec8 and Red1 peak coordinates [53]. We define axis coordinates as the intersection of at least two of the three marks (Fig 2c-2e). Percentages of overlap are indicated in the table below. b, Table of overlap between axis, DSBs, Tel1 and Spo11 peaks relative to each other’s (corresponding to percentages in Fig 2c). Numbers of identified peaks are indicated for the features in the first line relative to features in the first column. “Alone” in the bottom line refers to non-overlapping peaks with any features. d, e, Overlap between axis, DSBs and Tel1 peaks. Boxplots are comparing the overlap between real and shuffled distribution of Tel1 (c) or DSBs (d) peaks along the genome with the indicated feature. Ten independent random genomic region shuffling were generated. Centre, lower edge, and upper edge of the boxplot denote median, 25th, and 75th percentiles; lower end and higher end of the vertical lines denote 5th and 95th percentiles. Mann Whitney test p-values are indicated. (PDF) [file pgen.1011904.s004.pdf]

Figure S5

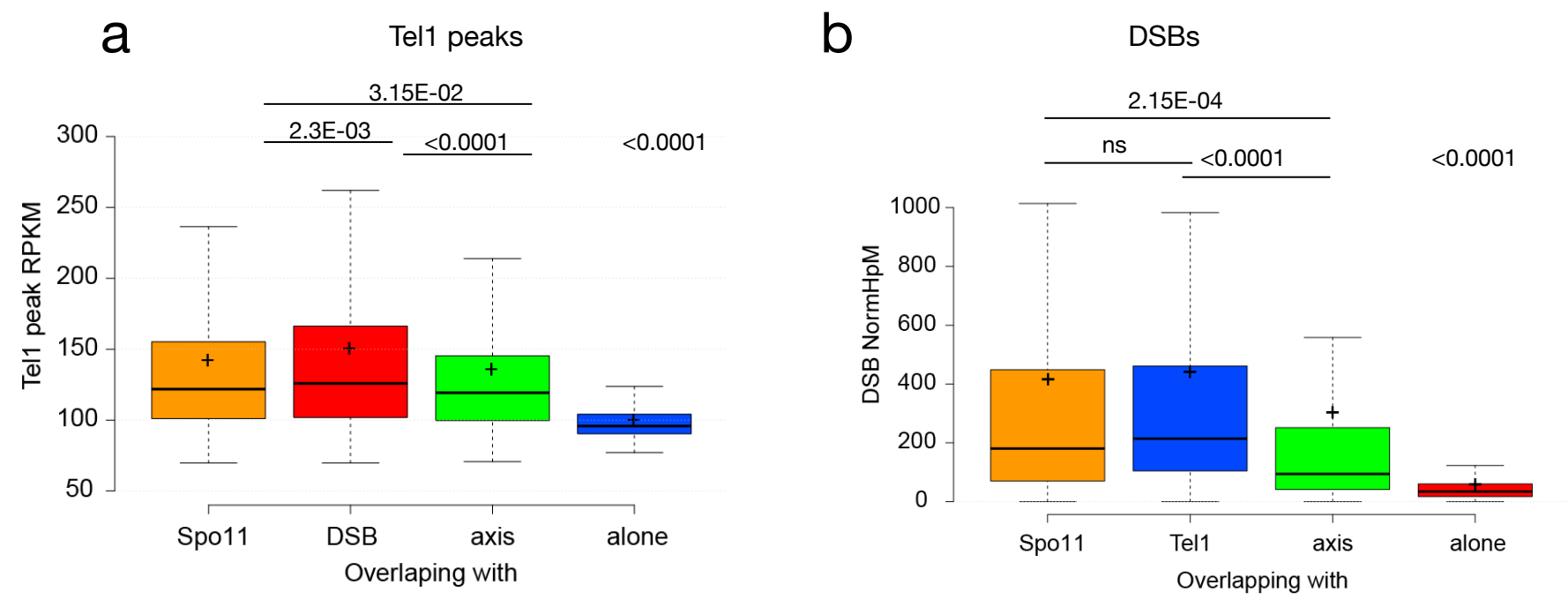

Supplement: S5 Fig — Tel1 and Spo11 peak strength was quantified by RPKM (see methods). DSBs peak strength was quantified by NormHpM (methods). The solid horizontal line represents the median, and the box encompasses the lower and upper quartiles. The vertical lines denote 5th and 95th percentiles. Mann Whitney test p-values are indicated. a, Tel1 peaks are distributed according to their overlap with Spo11, DSBs, axis, or with none of the above (‘alone’). Tel1 peaks not associated with other features (Spo11, DSBs or axis sites) are highly significantly weaker than all the others. b, DSBs peaks are distributed according to their overlap with Tel1, Spo11, axis, or with none of the above (‘alone’). DSBs peaks associated with axis sites, or not associated with other features (Tel1, Spo11 or axis sites) are significantly weaker than all others. (PDF) [file pgen.1011904.s005.pdf]

Figure S6

a

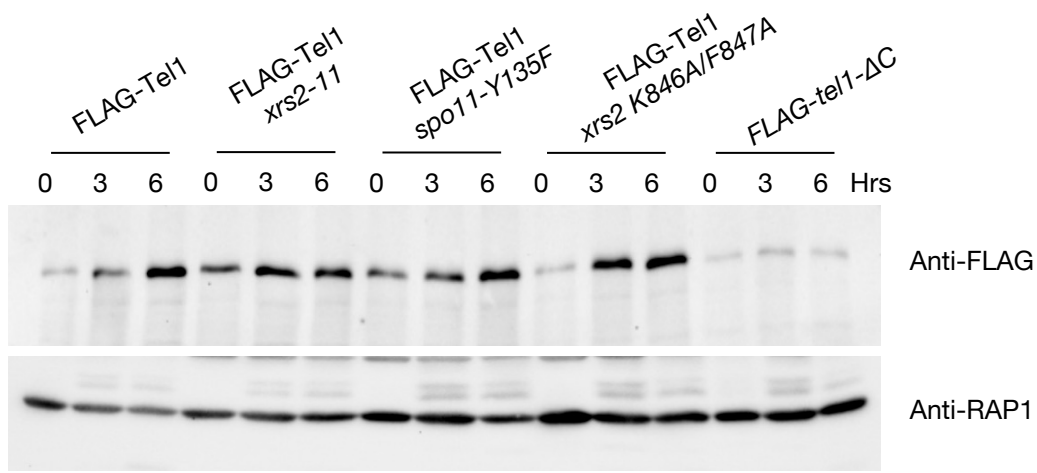

**b**

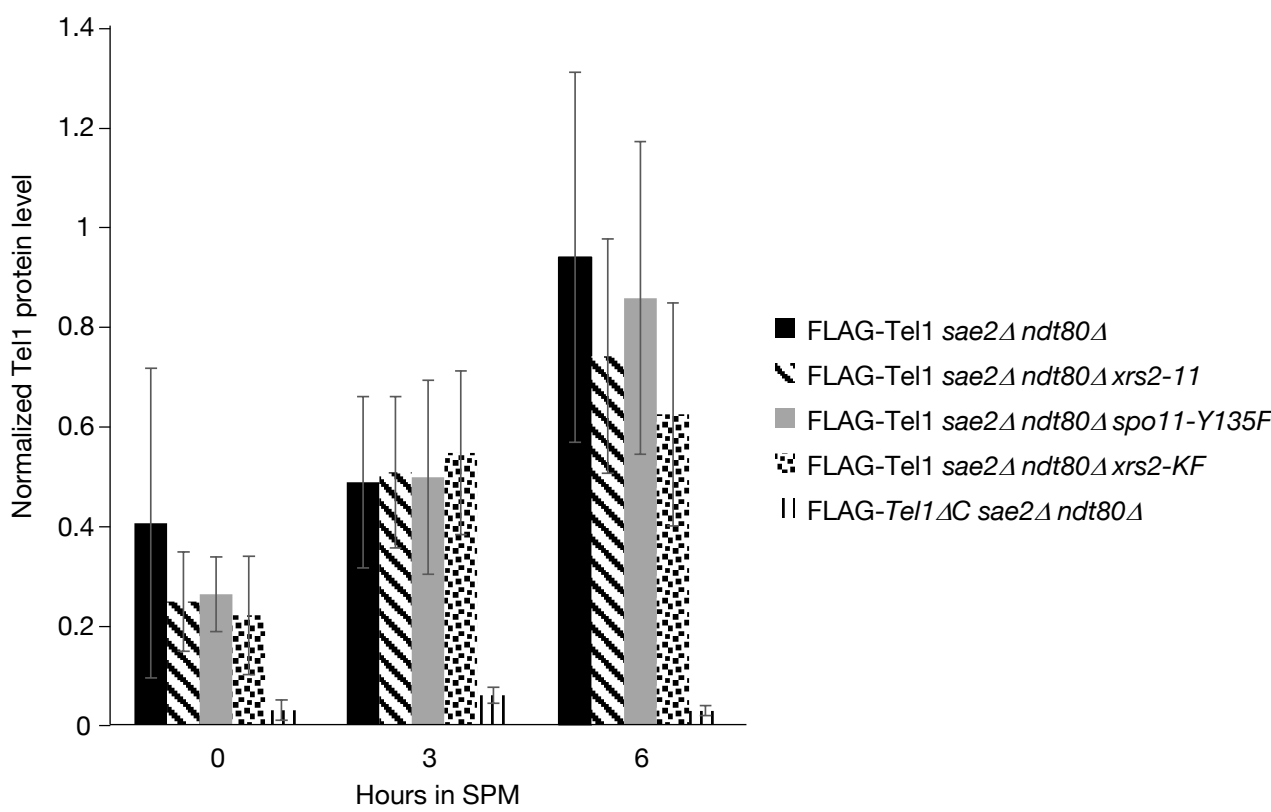

**C**

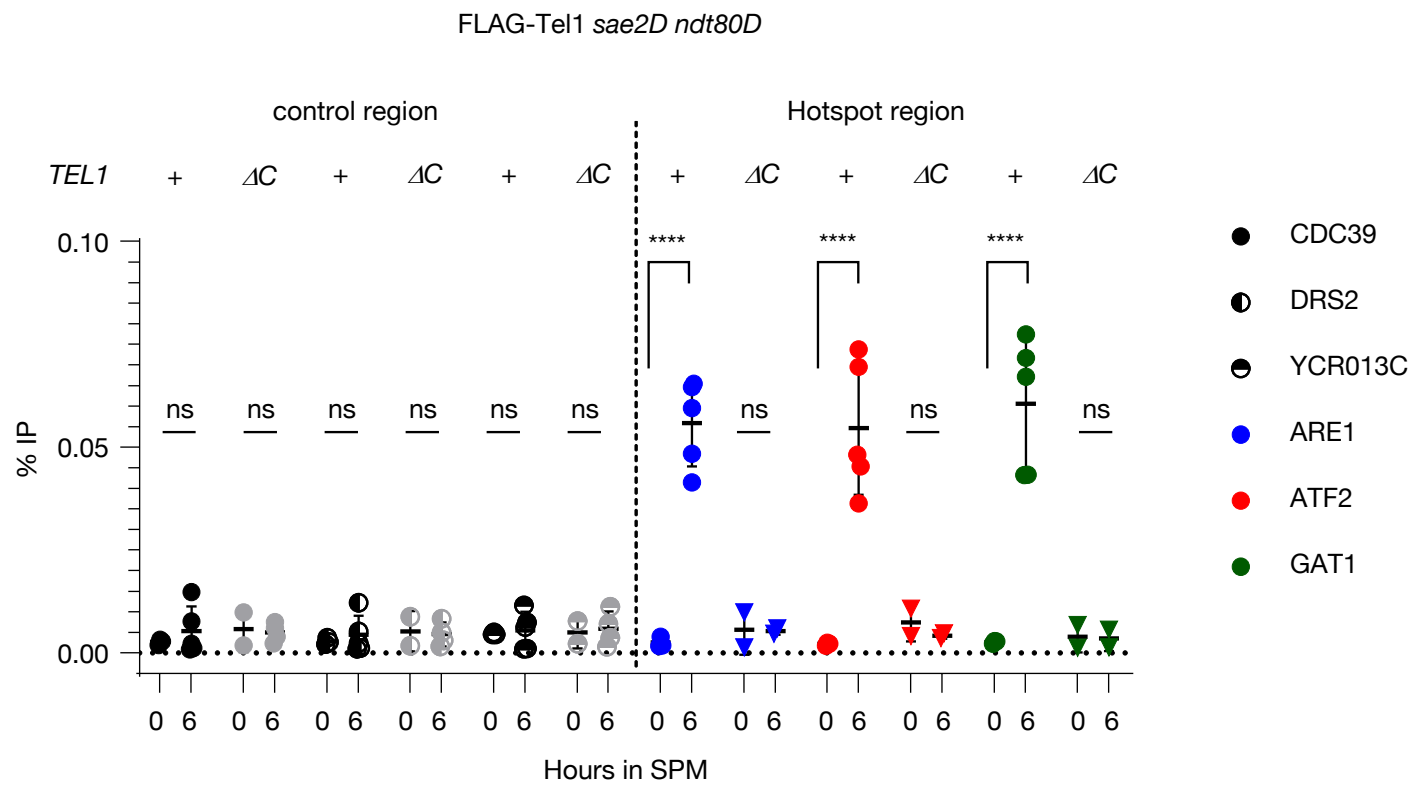

Supplement: S6 Fig — a, Immunoblot of meiotic TCA extracts at indicated time-points probed with anti-FLAG antibody (Tel1). Bottom panels: anti-RAP1 antibody. b, Quantification of immunoblots (n = 3) as per (a). Signal normalization was performed against RAP1 signal. c, FLAG-Tel1-ΔC recruitment to meiotic hotspots measured by ChIP-qPCR at the ARE1, ATF2 and GAT1 hotspot relative to three control sites (CDC39, DRS2 and YCR013C)in sae2Δ ndt80Δand sae2Δ ndt80Δ FLAG-tel1-ΔC strains. Error bars indicate SD from 3 (t 0 h) and 5 (t 6 h) independent experiments. P values are from paired ANOVA test between time 0 h and 6 h, corrected via Two-stage linear step-up procedure of Benjamini, Krieger and Yekutieli for multiple comparisons test. ns (not significant), ****, p < 0.0001. (PDF) [file pgen.1011904.s006.pdf]

Figure S7

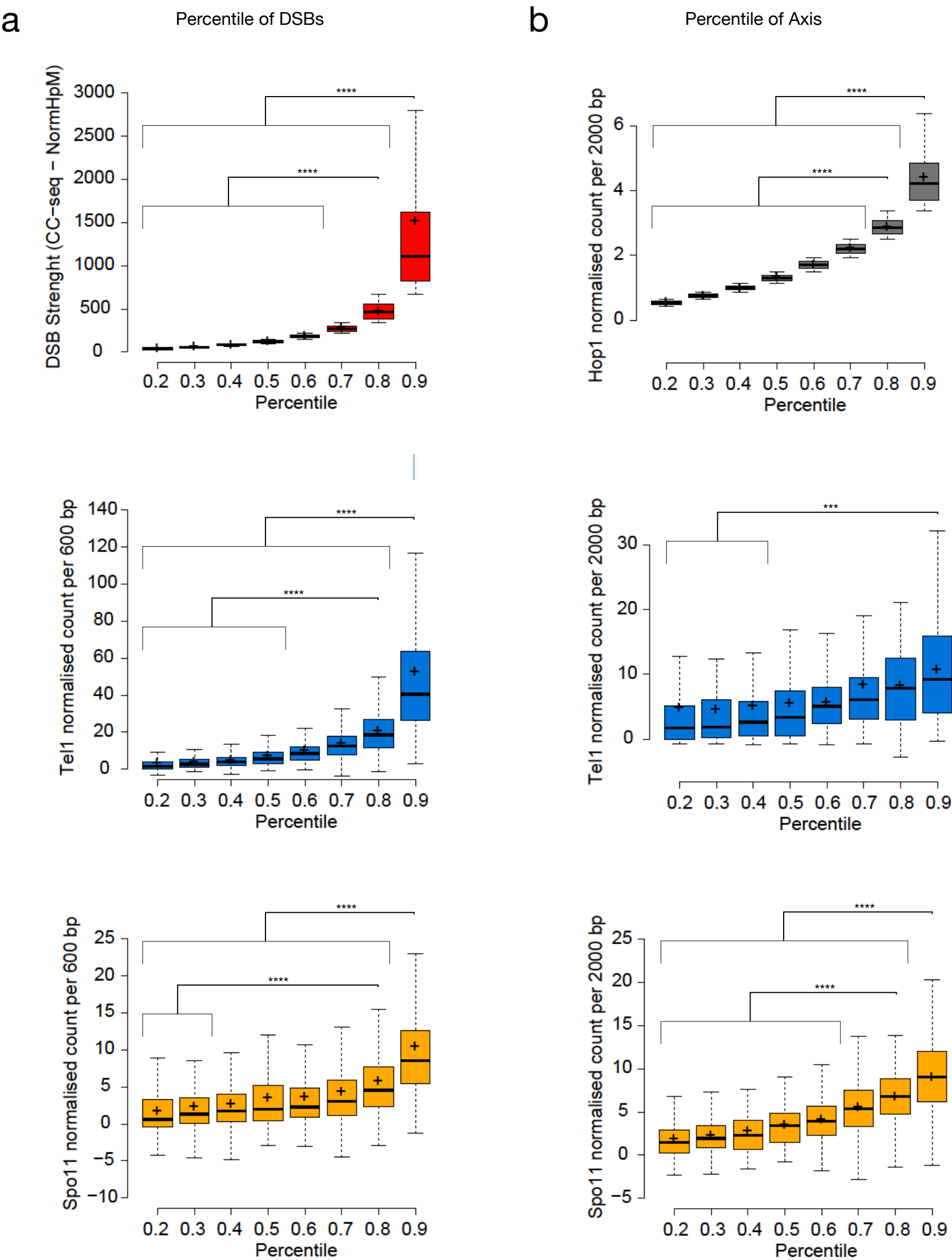

Supplement: S7 Fig — a, Boxplot of DSBs, Tel1 and Spo11 normalized counts per 600 bp (see Methods) partitioned by DSB strength percentile (NormHPM). b, Boxplot of Axis (Hop1), Tel1 and Spo11 normalized counts per 2kb (see Methods) partitioned by Hop1 strength percentile (RPKM). Mann Whitney test p-values are indicated. (PDF) [file pgen.1011904.s007.pdf]

Figure S8

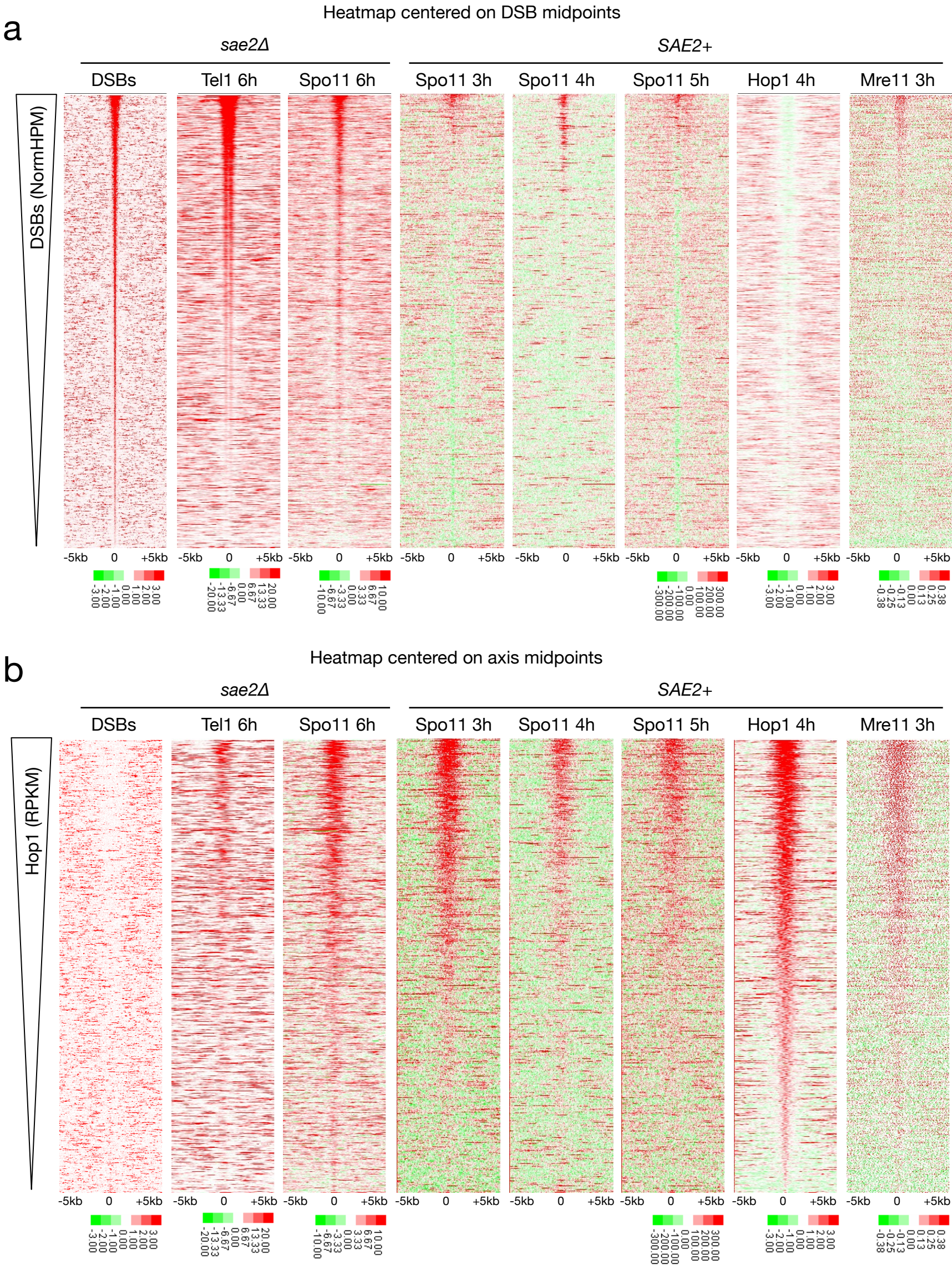

Supplement: S8 Fig — Regions are ordered according to DSBs strength (NormHpM) in all heatmaps presented in (a) or according to Hop1 ChIP-seq signal(53) (RPKM) in all heatmaps presented in (b). (PDF) [file pgen.1011904.s008.pdf]

Figure S9

a

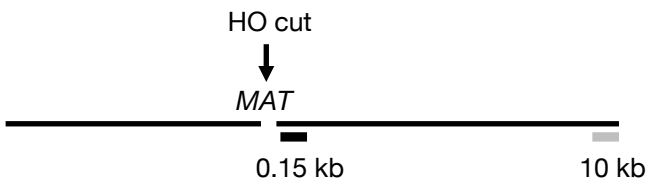

b

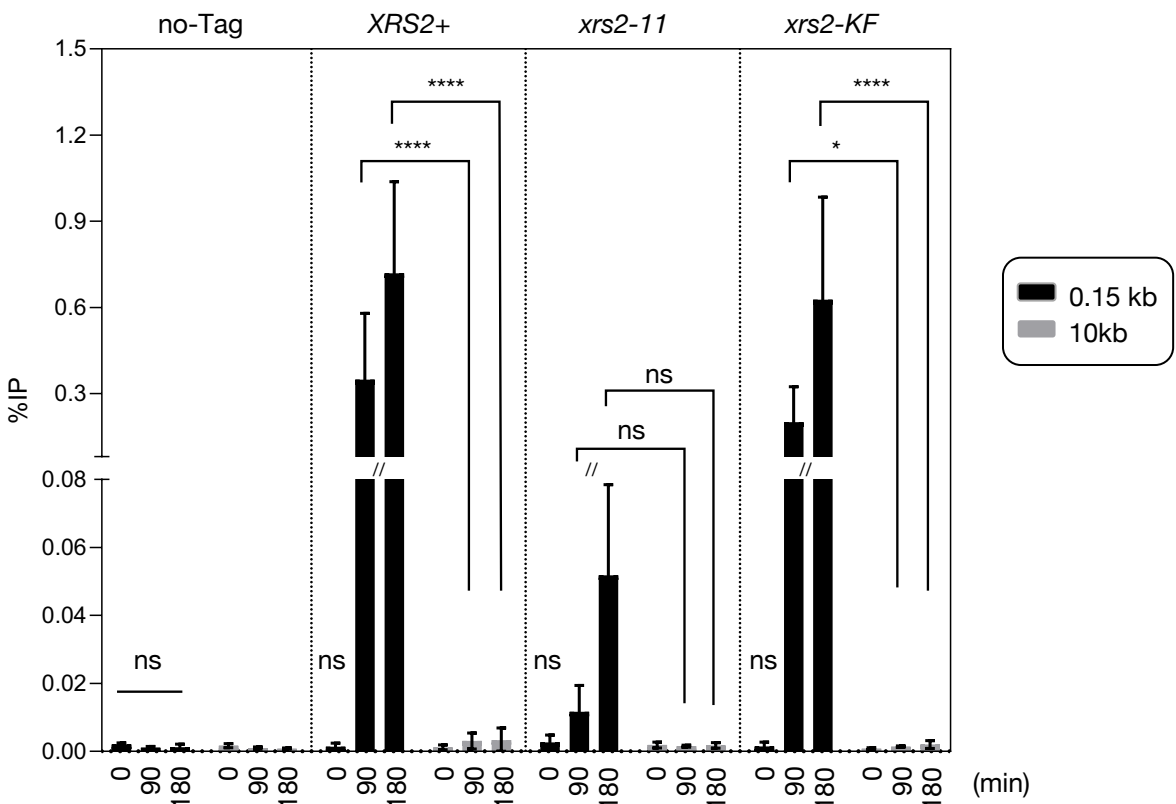

Supplement: S9 Fig — a, Diagram of the MAT (HO site) with the primer pairs to measure Tel1 binding by ChIP-qPCR. b, Tel1 recruitment to the Gal::HO break is dependent of the C-terminal part of Xrs2. Recruitment of FLAG-Tel1 was measured by ChIP-qPCR at 0’, 90’ and 180’ after induction of HO expression. Recruitment was measured at 0.15 kb (blue) and 10 kb (red) of the break. Bars represent the mean values of three independent experiments with error bars denoting s.d. (PDF) [file pgen.1011904.s009.pdf]

Figure S10

a

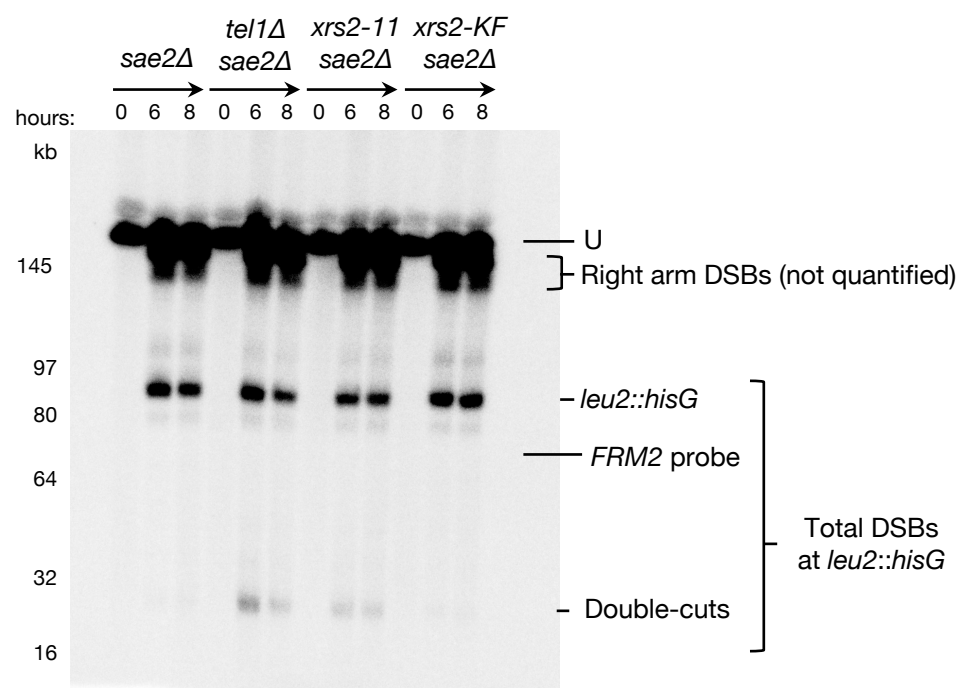

b

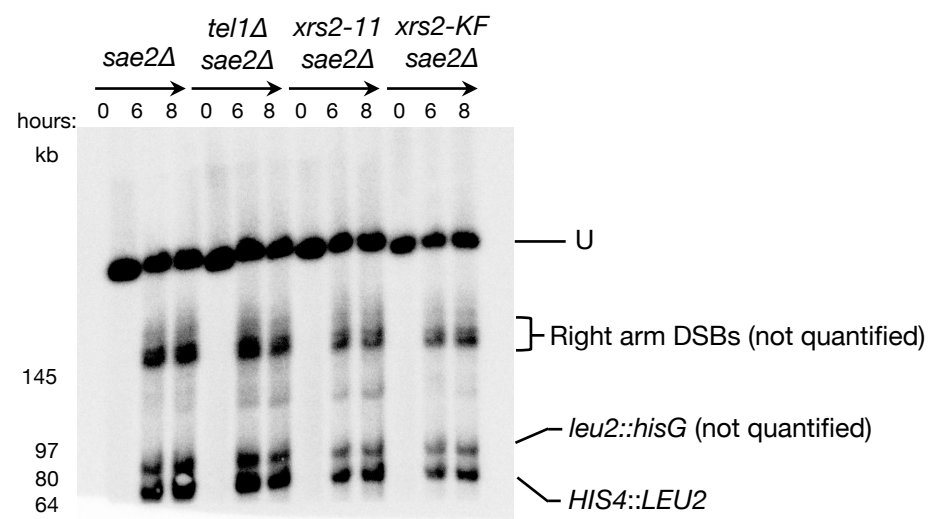

c

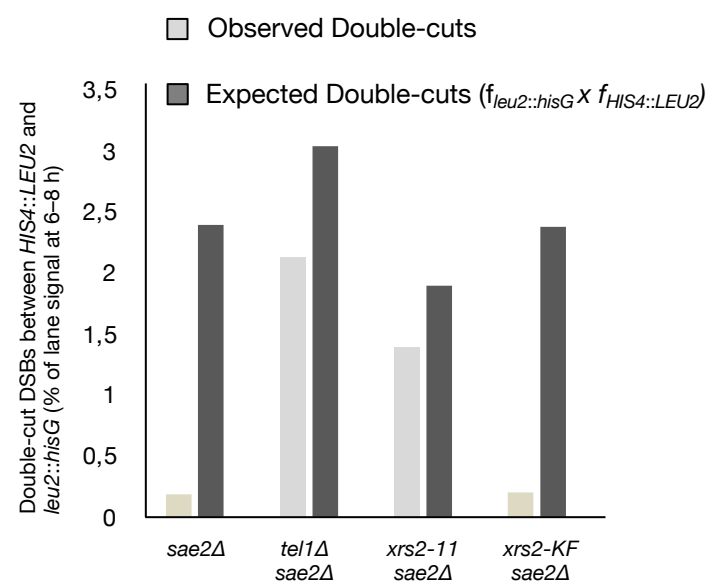

d

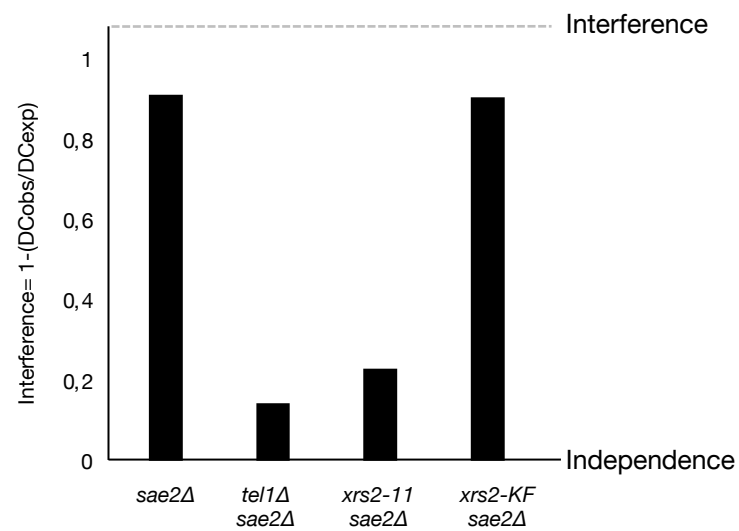

Supplement: S10 Fig — a, b, Genomic DNA isolated from the indicated time points after induction of meiosis was prepared in plugs and fractionated by PFGE, transferred to nylon membrane and hybridized with a FRM2 probe located between the HIS4::LEU2 and leu2::hisG hotspots (a), and with a CHA1 probe located on the left of HIS4::LEU2, on the left arm of chromosome III (b). Major double-cut signal is indicated by an asterisk. c, Quantification of observed double-cut signals and expected double-cuts from quantification in (a) and (b). Expected frequencies of double-cut molecules (as if forming independently) were calculated from measured single-cut frequencies at leu2::hisG (with FRM2 probe) and HIS4::LEU2 hotspots (with CHA1 probe). d, Interference values (I = 1-(observedDC/expectedDC), see Methods). (PDF) [file pgen.1011904.s010.pdf]

Figure S11

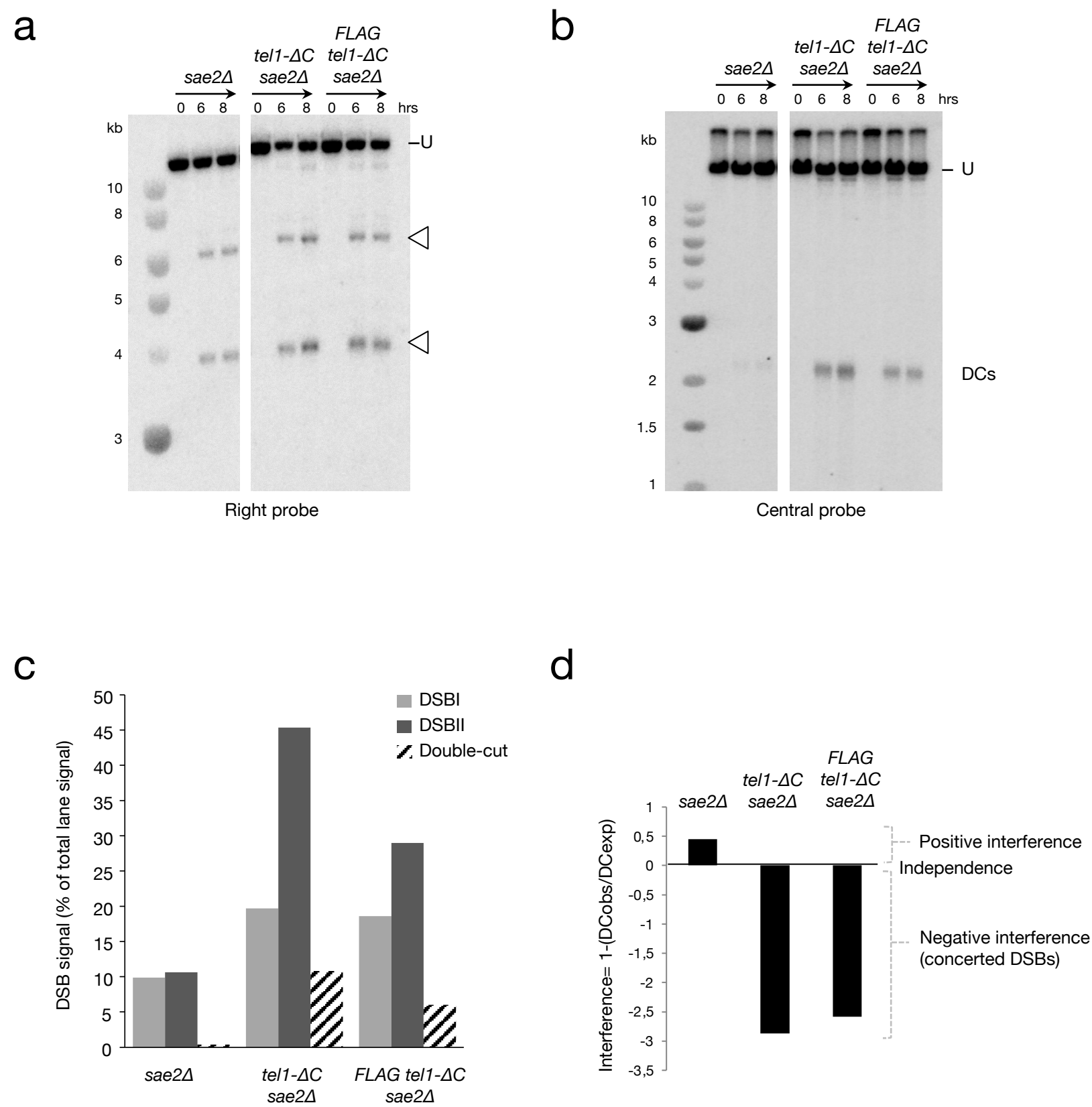

Supplement: S11 Fig — The tel1-ΔC mutation introduces a termination codon at the amino acid residue 2778, removing the amino acids 2778–2787. a, b, PstI-digested (a) or undigested (b) genomic DNA isolated from the indicated time points after induction of meiosis was fractionated by electrophoresis, transferred to nylon membrane and hybridized with probes as indicated (Right probe: MRX2, Central probe: LEU2). P, PstI-digested parental DNA; U, uncut parental DNA. DSB signals are marked with open triangles. Samples are parts of the same membrane that was cropped for presentation purpose. c, Quantification of DSBs and double-cut signals in (a) and (b). d, Interference values (I = 1-(observedDC/expectedDC), see Methods). (PDF) [file pgen.1011904.s011.pdf]
