# Supplementary material for: Tel1 is recruited at chromosomal loop/axis contact sites to modulate meiotic DNA double-strand breaks interference
Source: PLoS Genet. 2025 Nov 17;21(11):e1011904. doi: 10.1371/journal.pgen.1011904 (PMC12622785; doi:10.1371/journal.pgen.1011904)
Supplement: S1 Table — (PDF) [file pgen.1011904.s012.pdf]

Table S1 : List of strains used in this study

Strain	Mating type	Genotype
VG499	a/α	*ho::LYS2/", lys2/", ura3/", arg4-nsp/", leu2::hisG/", his4X::LEU2/", nuc1::LEU2/", sae2Δ::KanMX6/"*
VG739	a/α	*ho::LYS2/", lys2/", ura3/", arg4-nsp/", leu2::hisG/", his4X::LEU2/", nuc1::LEU2/"*
VG740	a/α	*ho::LYS2/", lys2/", ura3/", arg4-nsp/", leu2::hisG/", his4X::LEU2/", nuc1::LEU2/", FLAG-TEL1", sae2Δ::KanMX6"*
VG741	a/α	*ho::LYS2/", lys2/", ura3/", arg4-nsp/", leu2::hisG/", his4X::LEU2/", nuc1::LEU2/", FLAG-TEL1"*
VG906	a/α	*ho::LYS2/", lys2/", ura3/", arg4-nsp/3, leu2::hisG/", nuc1::LEU2, SPO11-His6-flag3-loxP-HphMX-loxP/", sae2Δ::KanMX6/"*
VG613	a/α	*ho::LYS2/', lys2/', ura3/', arg4-nsp/', leu2::hisG/', his4X::LEU2/', nuc1::LEU2/', xrs2CterΔ/", sae2Δ::KanMX6/"*
VG619	a/α	*ho::LYS2/', lys2/', ura3/', arg4-nsp/', leu2::hisG/', his4X::LEU2/', nuc1::LEU2/', xrs2 K846A/F847A::KANMX6/", sae2Δ::KanMX6/"*
VG749	a/α	*ho::LYS2/", lys2/", ura3/", arg4-nsp/", leu2::hisG/", his4X::LEU2/", nuc1::LEU2/", FLAG-TEL1/", spo11(Y135F)::KanMX4/", sae2Δ::KanMX6/"*
VG764	a/α	*ho::LYS2/", lys2/", ura3/", arg4-nsp/", leu2::hisG/", his4X::LEU2/", nuc1::LEU2/", FLAG-TEL1/", sae2Δ::KanMX6/", xrs2CterΔ::KanMX6/"*
VG838	a/α	*ho::LYS2/", lys2/", ura3/", arg4-nsp/", leu2::hisG/", his4X::LEU2/", nuc1::LEU2/", FLAG-TEL1/", sae2Δ::KanMX6/", xrs2 K846A/F847A::KANMX6/"*
VG948	a/α	*ho::LYS2/", lys2/", ura3/", arg4-nsp/", leu2::hisG/", his4X::LEU2/", nuc1::LEU2/", FLAG-tel1-ΔC::NatMX6/", sae2Δ::KanMX6/"*
VG1212	a/α	*ho::LYS2/', lys2/', ura3/', arg4-nsp/', leu2::hisG/', his4X::LEU2/', nuc1::LEU2/', FLAG-TEL1/', sae2Δ::KanMX6/', xrs2CterΔ::KanMX6/', ndt80::HphMX/'*
VG1238	a/α	*ho::LYS2/", lys2/", ura3/", arg4-nsp/", leu2::hisG/", his4X::LEU2/", nuc1::LEU2/", FLAG-TEL1/", sae2Δ::KanMX6/", ndt80::HphMX/"*
VG1582	a/α	*ho::LYS2/", lys2/", ura3/", arg4-nsp/", leu2::hisG/", his4X::LEU2/", nuc1::LEU2/", FLAG-TEL1/", sae2Δ::KanMX6/",spo11(Y135F) ::KanMX6/" ndt80::HphMX/"*
VG1590	a/α	*ho::LYS2/", lys2/", ura3/", arg4-nsp/", leu2::hisG/", his4X::LEU2/", nuc1::LEU2/", FLAG-TEL1/", sae2Δ::KanMX6/",xrs2 K846A/F847A ::KanMX6/" ndt80::HphMX/"*
VG1597	a/α	*ho::LYS2/", lys2/", ura3/", arg4-nsp/", leu2::hisG/", his4X::LEU2/", nuc1::LEU2/", FLAG-TEL1/", sae2Δ::KanMX6/",FLAG-tel1-ΔC ::NatMX6/" ndt80::HphMX/"*
VG1393	a	*hoΔ hmlΔ ::ADE1 hmrΔ ::ADE1 ade1-100 leu2,3-112 lys5 trp1::hisG ura3-52 ade3::GAL::HO*
VG1447	a	*hoΔ hmlΔ ::ADE1 hmrΔ ::ADE1 ade1-100 leu2,3-112 lys5 trp1::hisG ura3-52 ade3::GAL::HO FLAG-TEL1 xrs2-11*
VG1448	a	*hoΔ hmlΔ ::ADE1 hmrΔ ::ADE1 ade1-100 leu2,3-112 lys5 trp1::hisG ura3-52 ade3::GAL::HO FLAG-TEL1 xrs2-K846A,F847A*
