# Supplementary material for: Tel1 is recruited at chromosomal loop/axis contact sites to modulate meiotic DNA double-strand breaks interference
Source: PLoS Genet. 2025 Nov 17;21(11):e1011904. doi: 10.1371/journal.pgen.1011904 (PMC12622785; doi:10.1371/journal.pgen.1011904)
Supplement: S2 Table — (PDF) [file pgen.1011904.s013.pdf]

Table S2: List of primers used in this study

Xrs2-11_pFA6_F@2030
ATCACTAGAAATTATGTTCCGTTAAAAAAATACTCCAAAAAGGATACAACTACAAAATGGGTGA GGCGCGCCACTTCTAAA
Xrs2-11_pFA6_R@2627
TATCGAATGATAATGCAAAATATAATTTAATGAAATTGGAAATACTCGGAAAATTTATCAATCGATGAATTCGAG CTC
Xrs2-K846A/F847A_pFA6_F@2030
TGATGGCGACGACGACGATGACGACGGTCCG GCGGCTACGTTCAAAAGAAGAAAAGGATA TGA GGCGCGCCACTTCTAAA
Tel1-ΔC_NatMX6_F
GTAATGGACTAAGTGTAGAGTCTAGCGTACAAGATTTGATTCAGCAAGCCACGGATCCATCAAATTTGAGTtag tgaattcgcgccacttctaaataagc
Tel1d-ΔC_NatMX6_R
CATCGCATTGACTTTGTACATTACTTTTCGTATTTCTATAAACAAAAAAAAGAAGTATAAAGCATCTGCATAGCA Aactggatggcggcgttagtatcga

Primers Southern-blot probes

Probe		Forward Primer	Reverse Primer
MRX2	Chr III	HIS4_F@+5170<br>CGTGAAGTGGAACGATGCCC	HIS4_R@+5493<br>GCAACTGTTTCCAGCCTTCACC
LEU2	Chr III	LEU2_F<br>ATATACCATTCTAATGTCTGC	LEU2_R<br>AAGGATTTTCTTAACTTCTTCGGCG
FRM2	Chr III	FRM2_F@+27<br>GCTATTACAAACCGTCGTACCATC	FRM2_R@+645<br>CATCGCTGAGGTATCATTACTTCAT
CHA1	Chr III	CHA1_F@-9<br>ACCAGCGAGATGTCGATAGTCTAC	CHA1_R@+1052<br>TCTGGAAATATGAAATTGTCAGCG

Primers qPCR

Locus		Site	Forward Primer	Reverse Primer	Fragment size
CDC39	ChrIII	Control	CDC39_F@+3863<br>CGCTCCACCGATGACTCAAA	CDC39_R@+4014<br>CGACTGGGATGGCTGATTCA	151pb
MRE11	ChrXIII	Control	MRE11_F@+505<br>CCACTAAGTTAGCATTGTACGG	MRE11_R@+610<br>CTTCTCGCATAGTCGGTACTTC	105pb
DRS2	ChrI	Control	DRS2_F@+1454<br>GTACCCTCCAGGTAAAGGTACG	DRS2_R@+1278<br>ATGCGTAATGCTACTGCAACC	168bp

ATF2	ChrVII	Hotspot	ATF2@F+1548<br>GAATGGGAATCGTTCTGCAAGC	ATF2@R+1671<br>CACTGCTTGCCTTTTGTACGAG	123pb
ARE1	ChrIII	Hotspot	ARE1_F@-58<br>TCACGCAGGTGGTTGTTCAG	ARE1_R@+63<br>GGAATTGAGGCTGCGGATCTTA	121pb
GAT1	ChrVI	Hotspot	GAT1@F+25<br>CGCCCTTCCCCTGTTCTG	GAT1@R+165<br>AAATTCAAGTCCGGGTCGAGG	138pb
RRP7	ChrIII	Hotspot	RRP7_F@+15<br>CATTAGCGCCATGAAGAACGG	RRP7_R@+100<br>CGAAGCTTCCTGGCTTTTGG	85pb
YBP1	ChrII	Axis	YBP1_F@+1811<br>GGCAGATGCCAAGAAGAGTG	YBP1_R@+1962<br>CGGAAGTTTATCAGGTTCGACTG	151pb
GRR1	ChrX	Axis	GRR1_F@+3435<br>GCGTTCCTGATGCTTCATCC	GRR1_R@+3302<br>CCTGACCAGATGAGGAATCTCC	133pb
